# Psychoeducation for Suicidal Behaviors in Inpatient Settings: A Scoping Review

**DOI:** 10.3390/bs15081005

**Published:** 2025-07-23

**Authors:** Laura Fusar-Poli, Camilla Figini, Francesca Moioli, Caterina Marchesi, Ana Kovic, Pierluigi Politi, Natascia Brondino

**Affiliations:** 1 Department of Brain and Behavioral Sciences, University of Pavia, Via Bassi 21, 27100 Pavia, Italy; camilla.figini@unipv.it (C.F.); francesca.moioli01@universitadipavia.it (F.M.); ana.kovic01@universitadipavia.it (A.K.); pierluigi.politi@unipv.it (P.P.); natascia.brondino@unipv.it (N.B.); 2 Department of Mental Health and Addiction, Azienda Socio-Sanitaria Territoriale (ASST) Pavia, 27100 Pavia, Italy; caterina_marchesi@asst-pavia.it

**Keywords:** psychoeducation, suicide, self-injury, hospitalization, inpatient, prevention, review

## Abstract

(1) Background: Suicide is a worldwide leading cause of death among people with mental disorders. Psychoeducation is an integral component of mental health care that may offer patients valuable tools to understand their conditions, develop coping strategies, and engage more effectively in the treatment process. In the present scoping review, we aimed to summarize the evidence on the implementation of psychoeducational interventions in inpatient settings after suicide attempts. (2) Methods: In August 2024, we searched the Web of Knowledge (all databases), PsycINFO, and CINAHL databases following the PRISMA-ScR guidelines. We included original articles evaluating the effects of psychoeducational interventions for patients hospitalized in psychiatric settings after a suicide attempt. We provided a narrative synthesis of the study characteristics and the main findings of the included studies. (3) Results: We included five papers reporting the results of six studies, of which two were randomized controlled trials. Participants were diagnosed with diverse mental disorders, and interventions were generally short in the hospitalization phase, with follow-ups in the short or long term. Outcomes were focused on suicidal ideation, depressive symptoms, and general functioning, along with feasibility and acceptability of the intervention. Psychoeducational interventions were generally well accepted, but more evidence is needed to determine their efficacy. (4) Conclusions: Psychoeducational intervention in an inpatient psychiatric setting may be important for the prevention of future suicide attempts. Nevertheless, research on the topic is still scarce. Methodologically sound randomized controlled trials evaluating the long-term efficacy of psychoeducational interventions on suicide prevention are needed. Future research should also investigate the utility of psychoeducation in non-psychiatric inpatient settings.

## 1. Introduction

Suicide is among the top 20 causes of death worldwide. According to the World Health Organization (WHO), more than 720,000 people die from suicide every year worldwide and for every suicide, there are many more attempts at suicide (WHO, 2025). In Italy, there were more than 3800 suicides in 2021—6.2 suicides for every 100,000 inhabitants per year (ISTAT, 2024). Of note, suicide is the fourth leading cause of death among young people worldwide. A recent epidemiological analysis based on data from the WHO mortality database reported that suicidal rates are 2 to 5 times higher among young males than females. Moreover, while most European countries reported favourable patterns with declining rates, with the exception of the United Kingdom, rising suicide rates among young people were observed in the United States as well as in most Central Latin America and Australasia in the period between 1990 and 2020 (Bertuccio et al., 2024).

Research has suggested that a previous suicide attempt and suicidal ideation represent the strongest risk factors associated with suicide, with 6- to 16-fold increased risk (Favril et al., 2023; Ribeiro et al., 2016). Moreover, psychiatric disorders were associated with a greatly elevated risk of suicide mortality, with risk ratios in the range of 4–13 (Favril et al., 2023). Indeed, meta-analytic evidence has estimated a pooled suicide rate for people with mental disorders of 312.8 per 100,000 person-years worldwide, with suicide rates raising to 534.3 per 100,000 person-year when considering only people with major depression (Fu et al., 2023). Other factors associated with an increased risk of suicide are the presence of physical illnesses (e.g., cancer, epilepsy) and sociodemographic factors (e.g., unemployment, low education). Contact with the criminal justice system, state care in childhood, access to firearms, and parental death by suicide also increase the risk of suicide mortality (Favril et al., 2023).

The period following discharge from inpatient psychiatric care is recognised as an especially high-risk time for patients who have attempted suicide (Chung et al., 2017; Large et al., 2011; Bostwick et al., 2016; O’Connell et al., 2021). Inpatient psychiatric care for suicidality is typically focused on pharmacological needs and symptom stabilization, without necessarily addressing suicidality from a psychosocial perspective or examining the multifactorial causes and triggers the patient may have experienced (Olarte-Godoy et al., 2023). Most of the studies on psychosocial interventions for reducing suicidal rates and suicidal behaviors have been conducted in the psychiatric outpatient population (Witt et al., 2021a, 2021b). Conversely, research on psychosocial interventions in inpatient settings is scarce.

A meta-analysis of ten small pilot or feasibility randomized control trials (RCTs) has evaluated psychosocial interventions aimed at suicide reduction in psychiatric inpatients. Cognitive Behavioral Therapy (CBT) and Dialectical Behavioural Therapy (DBT) were implemented in the majority of the included studies. Nevertheless, according to the results, the interventions were no more effective than control treatments in reducing suicidality, depression, hopelessness or suicide attempts post-therapy and at follow-up (Yiu et al., 2021). More recently, a larger RCT has tested the effect of brief CBT in inpatient setting in addition to treatment as usual, showing a reduction of suicide reattempts and rate of readmissions in the six-month follow-up. Substance use disorder moderated the treatment effect on readmission rates (Diefenbach et al., 2024). Secondary analyses also showed a reduction in post-discharge emergency department utilization among participants without substance use disorders (Diefenbach et al., 2025). Psychoeducation is a non-pharmacological intervention encompassing systematic and structured didactic programs that provide patients, their families, and caregivers with information about a disorder and its treatment (Pedersen et al., 2024; Rummel-Kluge & Kissling, 2008; Powell et al., 2022; Rabelo et al., 2021). Psychoeducation is an integral component of mental health care, offering patients valuable tools to understand their conditions, develop coping strategies, and engage more effectively in their treatment process. Practically, psychoeducational interventions could include any program in which there is interaction between the information provider and the patient, with a focus on increasing knowledge by working on cognitive, affective, and/or psychomotor processes (Xia et al., 2011). The interventions may take place in small groups, one-on-one, or with family or caregivers. Although differing in organization and content, the programs should address the illness in a multidimensional way with a focus on familial, social, biological, and pharmacological perspectives. Psychoeducational interventions should be personalized to better serve the patient and their needs (Smith et al., 2010).

Implementing psychoeducational interventions in inpatient psychiatric settings might be particularly important for people who have attempted suicide for several reasons. First, psychoeducational programs can teach coping skills, crisis planning, and early warning signs, helping patients develop safer responses to distress (Stallman, 2018). Many suicide attempts occur during periods of emotional overwhelm or poor problem-solving. Psychoeducational interventions often include training in emotional regulation, stress management, and interpersonal effectiveness, which are protective against future crises (Iuso et al., 2022; Sökmen & Karaca, 2023). Psychoeducation may help patients to enhance their understanding of symptoms, triggers, and the course of mental disorder (Bossema et al., 2011). In turn, this can reduce confusion, shame, and self-blame, which are often intensified after a suicide attempt. Additionally, patients may feel ambivalent or resistant toward ongoing psychiatric care after attempting suicide. Psychoeducation helps them understand the importance of medication, therapy, and follow-up. This can lead to better engagement and adherence to treatment plans, reducing relapse risk (Iuso et al., 2023). The hospital stay is a critical window when patients are most vulnerable but also most accessible to interventions. Psychoeducation can provide structure, direction, and meaning during this period, facilitating a smoother transition to outpatient care. Finally, involving family members in psychoeducation can help them better understand the patient’s condition and provide more effective support. It can also help patients and families recognize when additional help is needed (Luciano et al., 2015; Roncone et al., 2007). This can improve the home environment and reduce conflict or misunderstanding, which are often contributors to emotional distress. 

Despite the potential relevance of implementing psychoeducation in inpatient settings, to the best of our knowledge, no reviews have systematically summarized the available literature focused on suicide. The aim of the present scoping review is thus to evaluate the acceptability and efficacy of psychoeducational interventions for patients hospitalized in psychiatric settings after a suicide attempt. 

## 2. Materials and Methods

### 2.1. Search Strategy

The present scoping review is part of a larger work aimed at identifying the effects of psychoeducational interventions in inpatient settings. In performing the scoping review, we followed the Preferred Reporting Items for Systematic Reviews and Meta-Analyses Extension for Scoping Reviews (PRISMA-ScR) guidelines (Tricco et al., 2018). A preliminary search for existing scoping reviews on the topic was conducted. In August 2024, we conducted systematic searches in Web of Knowledge (all databases), PsycINFO, and CINHAL. The search strings used are reported in the Supplementary Materials.

### 2.2. Selection Procedure

All records were extracted to Rayyan, an online tool specifically designed for the management of references while conducting systematic reviews (https://www.rayyan.ai/). Duplicates were detected and deleted. Two independent reviewers (C.M. and F.M.) initially screened the titles and abstracts and selected the studies to be included after checking the full text. In case of disagreement, the final decision was made with the help of a third reviewer (L.F.-P.).

Eligibility criteria were as follows: (1) Participants: individuals hospitalized in a psychiatric setting after a suicide attempt. (2) Intervention: psychoeducational interventions, defined as any form of therapeutic approach that provide individuals with information and education about their mental health conditions, symptoms, and treatment options, both manualized and non-manualized. (3) Comparison: any comparison (either another treatment or no comparison). (4) Outcomes: any outcome; (5) Study design: randomized controlled trials, longitudinal studies. We excluded (1) Studies written in languages other than English. (2) Studies including other psychosocial interventions along with psychoeducation. (3) Reviews, conference abstracts, case reports, and case studies.

### 2.3. Data Extraction and Synthesis

Relevant data on study characteristics, participants’ characteristics, interventions’ characteristics and description, outcome, and main findings were initially extracted. Any disagreement was solved through discussion with a third author (L.F.-P.) to reach a consensus. All the co-authors reviewed and discussed the resulting draft to provide a theoretical point of view.

## 3. Results

### 3.1. Search Results

**Figure 1:**
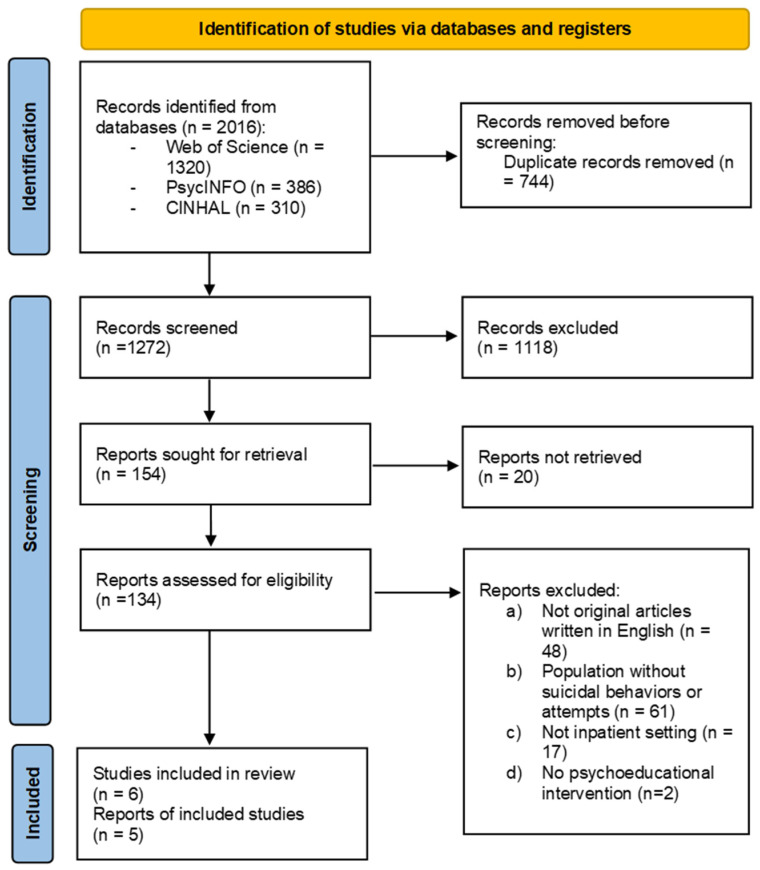
PRISMA flow diagram of the study selection process.

The PRISMA flow diagram of the study selection process is reported in Figure 1. 

Our literature search (i.e., on the broader topic of psychoeducation in psychiatry) identified a total of 244 publications. After removing duplicates, 183 titles and abstracts were screened. The full texts of 18 articles were then fully read for a more detailed evaluation. Finally, 5 papers were included in the scoping review. Characteristics of the included studies are presented in Table 1. 

### 3.2. Characteristics of Included Studies

Six studies reported in five papers (Amadeo et al., 2015; King et al., 2006; Bahlmann et al., 2022; Gebhardt et al., 2022; Yen et al., 2019) were included in the present scoping review. A summary of the relevant characteristics of the studies included in the review is presented in Table 1. Of note, one paper (Gebhardt et al., 2022) proposed a two-step study with two different datasets and is presented twice in the table. Papers were published between 2006 and 2022 and conducted in various countries, including three continents (America, Oceania, and Europe). More specifically, three studies reported in two papers were conducted in the United States, one in Germany, and one in French Polynesia. As for the study designs, two (Amadeo et al., 2015; King et al., 2006) were RCTs, adopting a between-groups design with randomization and comparing the active treatment to treatment as usual (TAU), while three studies (Bahlmann et al., 2022; Gebhardt et al., 2022; Yen et al., 2019) presented an open-label design in which treatment feasibility and outcomes were analysed on a single group without a control group for comparison.

### 3.3. Characteristics of Participants

Sample sizes ranged from 20 to 236 participants (for a total of 610 participants) with heterogeneous characteristics. Three studies (Amadeo et al., 2015; Bahlmann et al., 2022; Gebhardt et al., 2022) recruited an adult population, while other two studies (Yen et al., 2019; King et al., 2006) focused on adolescents. Participants’ diagnoses were widely variable, including mood disorders (Amadeo et al., 2015; Bahlmann et al., 2022; Yen et al., 2019), anxiety disorders (Amadeo et al., 2015), personality disorders (Amadeo et al., 2015), substance use disorder (Amadeo et al., 2015; Yen et al., 2019), obsessive-compulsive disorder (Bahlmann et al., 2022), bulimia nervosa (Bahlmann et al., 2022), and acute stress disorder (Bahlmann et al., 2022). The study by King et al. (2006) referred more generally to psychiatric disorders, while the study by Gebhardt et al. (2022) specifically recruited a population of veterans hospitalized in an inpatient psychiatric unit.

### 3.4. Characteristics and Description of the Interventions 

In terms of the type and content of the interventions offered to study participants, the studies reported a wide variability in both focus and delivery format. Individual interventions were chosen in three studies (Amadeo et al., 2015; Yen et al., 2019; Bahlmann et al., 2022) and group interventions in two studies (Gebhardt et al., 2022; King et al., 2006). Most of the proposed interventions consisted of a single session (Amadeo et al., 2015; Gebhardt et al., 2022), while brief series of four and five sessions were proposed by Yen et al. (2019) and Bahlmann et al. (2022), respectively. The duration of a single session could vary from 30 min to 2 h, with diverse content. Four studies provided follow-ups after the inpatient interventions (Bahlmann et al., 2022; Amadeo et al., 2015; King et al., 2006; Yen et al., 2019). Of note, the interventions implemented by Yen et al. (2019) and King et al. (2006) also proposed the involvement of external figures, such as peers or parents as a source of support.

Specific definitions of the interventions proposed in the included studies are reported in Table 1. The Brief Intervention and Contact (BIC; Amadeo et al., 2015) consisted of a psychoeducation session of one hour and a phone follow-up with 9 contacts within 18 months. 

The Relapse Prevention Intervention after Suicidal Event (RISE; Bahlmann et al., 2022) was a brief structured intervention specifically developed for patients admitted after a suicide attempt, aimed at reducing future suicidal events, improving coping strategies like increasing crisis expertise, developing actions for crisis management, and empowering the patient to manage suicidal ideation. The program was structured in five sessions: behavioral analysis, case conceptualization, psychoeducation and managing suicidal ideation, safety planning, and relapse prevention.

The Understanding Suicide Group (Gebhardt et al., 2022) consisted of a single session to understand the function of suicidal thoughts and behaviors and various factors contributing to individual suicide risk across the lifetime.

The Youth Nominated Support Team for Suicide (YST; Yen et al., 2019) was composed of psychoeducation sessions (1.5–2 h) designed to help understand the youth’s psychiatric disorders and treatment plan, suicide risk factors, strategies for communicating with adolescents, and emergency contact information. It was a manualized intervention conducted in groups, with the possibility to nominate one peer support person.

The Skills to Enhance Positivity in Suicidal Adolescents (STEP; Yen et al., 2019) was based on the Broaden and Build theory of emotion, focused on the function of positive emotions and three sets of skills (i.e., mindful meditation, gratitude, and savoring). The programme also included parents.

### 3.5. Outcomes

The outcomes of the interventions can be grouped into three assessment areas: (1) suicidal ideation, measured through clinical parameters like the number of suicides/repeated suicidal episodes, and structured scales, such as the Beck Scale for Suicide, the Columbia-Suicide severity Rating Scale (C-SSRS), and the Suicide Ideation Questionnaire (SIQ, SIQ-R); (2) depressive symptoms and general functioning, using measures like the Montgomery-Asberg Depression Rating Scale (MADRS), the Quick Inventory of Depressive Symptomatology (QIDS-SR), the Hopelessness Scale, the Reynolds Adolescent Depression Scale (RADS), and the Child and Adolescent Functional Assessment Scale (CAFAS); (3) feasibility and acceptability of the treatment, measured using general Likert scales or client satisfaction questionnaires. 

### 3.6. Main Findings

Amadeo et al. (2015) reported no statistically significant differences in non-fatal suicidal behaviors (*p* = 0.36) or suicide (*p* = 0.5) at 18-month follow-up between the groups that followed the psychoeducational intervention BIC and the group that received TAU alone.

After implementation of the RISE program in a longitudinal single-arm study, Bahlmann et al. (2022) reported a significant reduction of suicidal ideation (Cohen’s d = 0.75), hopelessness (Cohen’s d = 0.95), and decrease in the intensity of mental pain (Cohen’s d = 0.53) Additionally, there was a significant reduction in both clinician-rated (Cohen’s d = 0.79) and self-reported (Cohen’s d = 1.17) depressive symptoms, as well as in participants’ self-efficacy, that is the participant’s belief of coping with challenging situations (Cohen’s d = 0.93). At six-month follow-up, 89% of participants reported not having any suicide re-attempts, and most of the patients (94%) stated they have used the coping strategies learned during the program. 

The study published by Gebhardt et al. (2022) was designed in two phases, testing the acceptability and utility of a single-session psychoeducation group intervention in veterans. In the first pilot phase, the authors reported an elevated degree of treatment acceptability, with 98.5% of participants reporting no marked distress related to group participation. In the second phase, participants were asked to provide self-report feedback on the groups. 44% of participants were comfortable sharing their experiences, 24% uncomfortable, and 32% neutral. As for subjective distress, 49.3% reported no change in distress, 29.3 reported decreased distress, and 25.3 increased distress. According to the evaluations, the mostly appreciated characteristics were the provision of helpful information, the group discussion/format and the perception of a safe setting. Conversely, the participants reported that the sessions were too short, that they were uncomfortable discussing certain topics, and were not always satisfied with the scope of the information. Finally, participants suggested personalizing the intervention, inserting more group discussion (Gebhardt et al., 2022). 

King et al. (2006) detected a significant main effect for time in suicidal ideation (*p* < 0.001), but no time × group interaction. Interestingly, the authors reported a significant effect of sex, with a significant improvement in females attending the YST-1 treatment compared with males, with small-to-medium effect size (η^2^ = 0.06). There was no significant difference between groups in the proportion of adolescents who committed suicide attempts during the six-month period. Depressive symptoms improved over time, but there were no statistically significant differences according to the treatment group. Finally, mood-repeated impairment improved with time, without significant differences across interventions. Separate analyses conducted in males and females revealed a significant time x intervention group interaction for females only (η^2^ = 0.05 for intention-to-treat analysis, indicating a small effect) (King et al., 2006). 

Yen et al. (2019) implemented the STEP intervention, showing good feasibility and acceptability of the intervention. In the year after discharge, 5% of participants committed a suicide attempt, while 25% were readmitted due to suicide risk. Large effect sizes were observed for suicidal ideation from baseline to post-treatment (d = 1.07) and follow-up (d = 2.97), showing a decrease in suicidal ideation and low suicide attempt rates (Yen et al., 2019). 

## 4. Discussion

The present scoping review examined the literature on the effects of psychoeducation implemented after hospitalization for suicide attempts. Our review revealed a dearth of research on the topic, with only a few studies conducted up to the present date. In general, the findings of the included studies highlight key aspects related to both the potential benefits and challenges of implementing psychoeducation in inpatient settings. The role of psychoeducation in inpatient care can go far beyond the provision of basic information. It involves educating patients about their specific diagnoses and the medications prescribed, as well as introducing them to the different therapeutic options available to them. In addition, the evidence supports psychoeducational interventions, demonstrating the crucial role they can play in helping patients develop resilience and self-management skills, and maintaining satisfactory levels of motivation during the recovery process. By providing patients with the knowledge and tools to be active participants in their own care, psychoeducation supports not only understanding but also emotional and psychological well-being throughout the treatment process.

Previous research has shown how psychoeducational interventions, whichever the setting, can help reducing symptoms, improving adherence, and promoting recovery by providing patients the knowledge and skills necessary to manage their mental health challenges (Olarte-Godoy et al., 2023; Rummel-Kluge & Kissling, 2008; Smith et al., 2010; Xia et al., 2011). In addition, psychoeducation can alleviate the feelings of isolation and insecurity by increasing patients’ understanding and normalizing their struggles. In the context of suicidality, psychoeducation implemented in an inpatient setting may have significant potential as a strategic treatment for patients at high risk of suicide, as it can serve as an effective tool to help patients navigate the transition into the vulnerable post-discharge period. Indeed, a reduction in symptoms linked to suicidal ideation has been reported by the included studies, particularly during short-term follow-up periods.

The studies included in this review suggest that psychoeducation can provide patients with the necessary coping strategies, ultimately contributing to their long-term recovery and reducing the likelihood of suicidal behaviour in the critical period after discharge (Bahlmann et al., 2022; Olarte-Godoy et al., 2023). These improvements suggest that psychoeducation could be a valuable component of a comprehensive approach to suicidality prevention in inpatient settings, supporting both immediate and longer-term mental health. The effectiveness of such interventions is often measured by their feasibility and acceptability, which can be expressed through indicators like low dropout rates or positive patient feedback. 

To our knowledge, this is the first review to systematically summarize the evidence on psychoeducational interventions for people with mental disorders in inpatient settings. Nevertheless, some limitations should be mentioned. A major limitation is that the review was conducted without a pre-registered study protocol. Second, although we conducted a comprehensive literature search in compliance with the PRISMA-ScR guidelines (Tricco et al., 2018), we did not consult the grey literature and excluded conference abstracts and studies written in languages other than English. Third, the scope of our review was limited to psychoeducation, while other types of psychosocial interventions were not examined. Finally, we did not conduct a systematized and critical quality assessment of the included studies.

Specific limitations of the studies included should also be taken into account when interpreting the results of our review. First of all, the generalisability of findings in many studies is limited by several factors, such as the small sample sizes (Bahlmann et al., 2022; Yen et al., 2019; Gebhardt et al., 2022), or the lack of methodologically robust designs with a significant absence of control groups in non-controlled clinical trials (Bahlmann et al., 2022; Yen et al., 2019; Gebhardt et al., 2022) which reduces the reliability of results and makes it difficult to isolate the effect of psychoeducational interventions from other confounding factors. Target populations, such as inpatients with heterogeneous psychiatric disorders and different age groups (adolescents, adults, and veterans), further complicate comparisons of the studies. Differences in intervention format, structure, and duration, such as single versus multi-session programmes and individual versus group approaches, introduce additional variability. Furthermore, short follow-up periods, as seen in studies such as Yen et al. (2019), with follow-up periods of 1–3 months post-intervention, and Gebhardt et al. (2022), with immediate follow-up, make it difficult to draw conclusions about the long-term effects of psychoeducational interventions. Longer-term studies, such as Amadeo et al. (2015), which provide data from an 18-month follow-up, are rare, leading to an underestimation of the importance of assessing outcomes over time. In addition, self-reported outcome measures may introduce the potential for response bias, such as under-reporting due to stigma. Moreover, reported outcomes vary widely across studies, with studies such as Amadeo et al. (2015) finding no significant effects, King et al. (2006) finding effects limited to the subgroup of women, and Bahlmann et al. (2022), Yen et al. (2019) and Gebhardt et al. (2022) reporting positive effects on suicidal ideation and program feasibility. Ultimately, as pointed out in the study by Amadeo et al. (2015), TAU alone may provide substantial psychological support, potentially obscuring the additional benefits of the interventions.

Although this was not a focus of the present review, future research should also consider the implementation of psychoeducational intervention in non-psychiatric hospital settings, such as emergency rooms and other hospital services where people are hospitalized just after a suicide attempt (e.g., intensive care unit, traumatology, etc.). Environmental modifications and education of health care professionals appear to be the most promising strategies to reduce suicide-related mortality among inpatients (Navin et al., 2019). Psychoeducation can be delivered by different health professionals, and consultation-liaison psychiatrists may represent important figures for the education of health professionals working in critical care settings. 

## 5. Conclusions

In a context in which suicide is the leading cause of death among 15–29-year-old age range (WHO, 2025), it is becoming increasingly essential to implementing psychosocial interventions like psychoeducation, both for preventive and therapeutic purposes. Psychoeducation is a low-cost intervention which could be easily implemented by different health professionals also in the hospital setting.

The findings of the present scoping review are promising and indicate that psychoeducation interventions are feasible and could serve as complementary approaches for the prevention and treatment of suicidal behaviours alongside pharmacological treatments. Addressing the study limitations in future research may enhance the quality and applicability of findings. Future studies should establish a clear structure for psychoeducational interventions with standardised, evidence-based protocols that are adaptable to diverse clinical environments. Emphasis should be placed on evaluating the long-term effectiveness of these interventions in reducing the recurrence of suicidal behaviors and improving patient engagement in follow-up care. Additionally, multicenter and multinational studies should explore how to tailor interventions to individual needs, taking into account cultural, developmental, and diagnostic differences. Systematic reviews and meta-analyses on the effectiveness of psychoeducational interventions in psychiatric settings, along with RCTs conducted with robust methodological approaches, may help investigate the comparative efficacy of psychoeducational interventions in addition to standard treatment in different target populations to develop specific intervention guidelines.

## Figures and Tables

**Table 1:** Characteristics of the included studies.

Study	Country	Design	N Participants	Mean Age (Range)	N Males (%)	Suicidal Behavior	Type of Interventions	N Sessions (Duration, min)	Post-Discharge Follow-Up	Individual/Group	Presence of a Peer or a Family Member	Control	Outcome (Measure)	Results
(Amadeo et al., 2015)	French Polynesia	RCT	200	32.24 (NR)	67 (35.26)	A	BIC + TAU	1 (60)	9 follow-up contacts (phone calls at 1, 2, 4, 7,11 week(s), and 4, 6, 12, 18 months)	I	N	TAU only	N of suicides and repeated non-fatal suicidal behavior (NFSB)	No statistically significant difference in the frequency of suicidal behavior (suicides and repeated NFSB) between TAU + BIC and TAU only.
(Bahlmann et al., 2022)	Germany	Longitudinal, single arm	20	35.6 (NR)	11 (55)	A	RISE + TAU	5 (50–60) session 3 focused on psychoeducation	Phone call after 6 months	I	N	N/A	Feasibility and acceptance, suicidal ideation (BSS), depressive symptoms (MADRS, QIDS-SR), hopelessness (BHS), self-efficacy (SWE)	Feasible intervention, highly accepted by the patients. Significant reduction in BSS, but no significant changes in SI intensity or in the intent to act on SI, significant decrease in the intensity of mental pain. Significant reduction in depressive symptoms and hopelessness, increase in self-efficacy.
(Gebhardt et al., 2022) (phase I)	United States	Longitudinal, single arm	56	46.11 (NR)	NR	A, I	Understanding suicide	1 (50–60)	No	G	N	N/A	Acceptability (Likert scales), tolerability (charts review, Likert scales)	Good acceptability, no marked distress following group participation.
(Gebhardt et al., 2022) (phase II)	United States	Longitudinal, single arm	78	48.11 (NR)	68 (87)		Understanding suicide	1 (50–60)	No	G	N	N/A	Acceptability (Likert scales), tolerability (Likert scales), group objectives (Likert scales)	Participants found group content new and useful; better understanding of STB and contributing factors, better identification of strategies to target STB; minimal changes in distress; more open to discuss STB with others.
(King et al., 2006)	United States	RCT	289	15.3 (12–17)	92 (31.8)	A, I	YST-1 + TAU	1 (90–120)	6 months	G	Yes	TAU only	Suicidal ideation (SIQ-R), depression (RADS), internalizing symptoms (YSR), mood/self-harm (CAFAS)	Improvement of SI and mood-related functional impairment only in females.
(Yen et al., 2019)	United States	Longitudinal, single arm	20	15,9 (12–18)	5 (25)	A, I	STEP	4, of which 1 with family member (30–45)	1 month,3 months, 1 year	I	Yes	N/A	Suicidal behaviors (C-SSRS), suicidal ideation (SIQ), satisfaction of participants and parents (CSQ)	Good feasibility, 1 suicide attempt, and 5 readmission for suicidality in the following 6 months

Legend: BHS: Beck Hopelessness Scale; BIC: Brief Intervention and Contact; BSS: Beck Scale for Suicide Ideation; CAFAS: Child and Adolescent Functional Assessment Scale; C-SSRS: Columbia Suicide Severity Rating Scale; CSQ: Client Satisfaction Scale; MADRS: Montgomery-Åsberg Depression Rating Scale; N/A: Not Applicable; NFSB: Non-fatal Suicidal Behavior; QIDS-SR: Quick Inventory of Depressive Symptomatology-Self Rating; RADS: Reynolds Adolescent Depression Scale; RCT: Randomized Controlled Trial; RISE: Relapse Prevention Intervention after Suicidal Event; SI: Suicidal Ideation; SIQ: Suicidal Ideation Questionnaire; STB: suicidal thoughts and behaviors; STEP: Skills to Enhance Positivity; SWE: General Self-Efficacy Scale; TAU: Treatment as Usual; YSR: Youth Self Report; YST: Youth Nominated Support Team.

## Data Availability

Not applicable.

## Supplementary Materials

The following supporting information can be downloaded at: https://www.mdpi.com/article/10.3390/bs15081005/s1, Word document S1: Search String.docx.

## References

[B1-behavsci-15-01005] Amadeo S., Rereao M., Malogne A., Favro P., Nguyen N. L., Jehel L., Milner A., Kolves K., De Leo D. (2015). Testing brief intervention and phone contact among subjects with suicidal behavior: A randomized controlled trial in French Polynesia in the frames of the world health organization/suicide trends in at-risk territories study. Mental Illness.

[B2-behavsci-15-01005] Bahlmann L., Lübbert M. B. J. S., Sobanski T., Kastner U. W., Walter M., Smesny S. W., Wagner G. (2022). Relapse Prevention Intervention after Suicidal Event (RISE): Feasibility study of a psychotherapeutic short-term program for inpatients after a recent suicide attempt. Frontiers in Psychiatry.

[B3-behavsci-15-01005] Bertuccio P., Amerio A., Grande E., La Vecchia C., Costanza A., Aguglia A., Berardelli I., Serafini G., Amore M., Pompili M., Odone A. (2024). Global trends in youth suicide from 1990 to 2020: An analysis of data from the WHO mortality database. EClinicalMedicine.

[B4-behavsci-15-01005] Bossema E. R., de Haar C. A., Westerhuis W., Beenackers B. P., Blom B. C., Appels M. C., van Oeveren C. J. (2011). Psychoeducation for patients with a psychotic disorder: Effects on knowledge and coping. The Primary Care Companion for CNS Disorders.

[B5-behavsci-15-01005] Bostwick J. M., Pabbati C., Geske J. R., McKean A. J. (2016). Suicide attempt as a risk factor for completed suicide: Even more lethal than we knew. American Journal of Psychiatry.

[B6-behavsci-15-01005] Chung D. T., Ryan C. J., Hadzi-Pavlovic D., Singh S. P., Stanton C., Large M. M. (2017). Suicide rates after discharge from psychiatric facilities: A systematic review and meta-analysis. JAMA Psychiatry.

[B7-behavsci-15-01005] Diefenbach G. J., Collett S., Black S., Rudd M. D., Gueorguieva R., Tolin D. F. (2025). The effect of inpatient brief cognitive-behavioral therapy for suicide prevention on post-discharge emergency department utilization: Secondary analysis of a randomized clinical trial. General Hospital Psychiatry.

[B8-behavsci-15-01005] Diefenbach G. J., Lord K. A., Stubbing J., Rudd M. D., Levy H. C., Worden B., Sain K. S., Bimstein J. G., Rice T. B., Everhardt K., Gueorguieva R., Tolin D. F. (2024). Brief cognitive behavioral therapy for suicidal inpatients: A randomized clinical trial. JAMA Psychiatry.

[B9-behavsci-15-01005] Favril L., Yu R., Geddes J. R., Fazel S. (2023). Individual-level risk factors for suicide mortality in the general population: An umbrella review. The Lancet. Public Health.

[B10-behavsci-15-01005] Fu X. L., Qian Y., Jin X. H., Yu H. R., Wu H., Du L., Chen H. L., Shi Y. Q. (2023). Suicide rates among people with serious mental illness: A systematic review and meta-analysis. Psychological Medicine.

[B11-behavsci-15-01005] Gebhardt H. M., Ammerman B. A., Carter S. P., Stanley I. H. (2022). Understanding suicide: Development and pilot evaluation of a single-session inpatient psychoeducation group. Psychological Services.

[B12-behavsci-15-01005] (2024). *Istituto Nazionale di Statistica (ISTAT)*.

[B13-behavsci-15-01005] Iuso S., Severo M., Trotta N., Ventriglio A., Fiore P., Bellomo A., Petito A. (2023). Improvements in treatment adherence after family psychoeducation in patients affected by psychosis: Preliminary findings. Journal of Personalized Medicine.

[B14-behavsci-15-01005] Iuso S., Severo M., Ventriglio A., Bellomo A., Limone P., Petito A. (2022). Psychoeducation reduces alexithymia and modulates anger expression in a school setting. Children.

[B15-behavsci-15-01005] King C. A., Kramer A., Preuss L., Kerr D. C. R., Weisse L., Venkataraman S. (2006). Youth-nominated support team for suicidal adolescents (version 1): A randomized controlled trial. Journal of Consulting and Clinical Psychology.

[B16-behavsci-15-01005] Large M., Sharma S., Cannon E., Ryan C., Nielssen O. (2011). Risk factors for suicide within a year of discharge from psychiatric hospital: A systematic meta-analysis. The Australian and New Zealand Journal of Psychiatry.

[B17-behavsci-15-01005] Luciano M., Del Vecchio V., Sampogna G., De Rosa C., Fiorillo A. (2015). Including family members in psychoeducation for bipolar disorder: Is it worth it?. Bipolar Disorders.

[B18-behavsci-15-01005] Navin K., Kuppili P. P., Menon V., Kattimani S. (2019). Suicide prevention strategies for general hospital and psychiatric inpatients: A narrative review. Indian Journal of Psychological Medicine.

[B19-behavsci-15-01005] O’Connell P. H., Durns T., Kious B. M. (2021). Risk of suicide after discharge from inpatient psychiatric care: A systematic review. International Journal of Psychiatry in Clinical Practice.

[B20-behavsci-15-01005] Olarte-Godoy J., Jack S. M., Campbell K., Halladay J., Cleverley K., McGillion M., Links P. (2023). Psychosocial interventions addressing suicidality in inpatient psychiatry: A scoping review protocol. JBI Evidence Synthesis.

[B21-behavsci-15-01005] Pedersen H., Skliarova T., Pedersen S. A., Gråwe R. W., Havnen A., Lara-Cabrera M. L. (2024). Psychoeducation for adult ADHD: A scoping review about characteristics, patient involvement, and content. BMC Psychiatry.

[B22-behavsci-15-01005] Powell L. A., Parker J., Weighall A., Harpin V. (2022). Psychoeducation intervention effectiveness to improve social skills in young people with ADHD: A meta-analysis. Journal of Attention Disorders.

[B23-behavsci-15-01005] Rabelo J. L., Cruz B. F., Ferreira J. D. R., Viana B. M., Barbosa I. G. (2021). Psychoeducation in bipolar disorder: A systematic review. World Journal of Psychiatry.

[B24-behavsci-15-01005] Ribeiro J. D., Franklin J. C., Fox K. R., Bentley K. H., Kleiman E. M., Chang B. P., Nock M. K. (2016). Self-injurious thoughts and behaviors as risk factors for future suicide ideation, attempts, and death: A meta-analysis of longitudinal studies. Psychological Medicine.

[B25-behavsci-15-01005] Roncone R., Mazza M., Ussorio D., Pollice R., Falloon I. R., Morosini P., Casacchia M. (2007). The questionnaire of family functioning: A preliminary validation of a standardized instrument to evaluate psychoeducational family treatments. Community Mental Health Journal.

[B26-behavsci-15-01005] Rummel-Kluge C., Kissling W. (2008). Psychoeducation in schizophrenia: New developments and approaches in the field. Current Opinion in Psychiatry.

[B27-behavsci-15-01005] Smith D., Jones I., Simpson S. (2010). Psychoeducation for bipolar disorder. Advances in Psychiatric Treatment.

[B28-behavsci-15-01005] Sökmen Z., Karaca S. (2023). The effect of self-regulation based cognitive psychoeducation program on emotion regulation and self-efficacy in children diagnosed with attention deficit hyperactivity disorder. Archives of Psychiatric Nursing.

[B29-behavsci-15-01005] Stallman H. M. (2018). Coping planning: A patient-centred and strengths-focused approach to suicide prevention training. Australasian Psychiatry: Bulletin of Royal Australian and New Zealand College of Psychiatrists.

[B30-behavsci-15-01005] Tricco A. C., Lillie E., Zarin W., O’Brien K. K., Colquhoun H., Levac D., Moher D., Peters M. D. J., Horsley T., Weeks L., Hempel S., Akl E. A., Chang C., McGowan J., Stewart L., Hartling L., Aldcroft A., Wilson M. G., Garritty C., Straus S. E. (2018). PRISMA Extension for Scoping Reviews (PRISMA-ScR): Checklist and explanation. Annals of Internal Medicine.

[B31-behavsci-15-01005] Witt K. G., Hetrick S. E., Rajaram G., Hazell P., Taylor Salisbury T. L., Townsend E., Hawton K. (2021a). Interventions for self-harm in children and adolescents. The Cochrane Database of Systematic Reviews.

[B32-behavsci-15-01005] Witt K. G., Hetrick S. E., Rajaram G., Hazell P., Taylor Salisbury T. L., Townsend E., Hawton K. (2021b). Psychosocial interventions for self-harm in adults. The Cochrane Database of Systematic Reviews.

[B33-behavsci-15-01005] (2025). *World Health Organization (WHO)*.

[B34-behavsci-15-01005] Xia J., Merinder L. B., Belgamwar M. R. (2011). Psychoeducation for schizophrenia. The Cochrane Database of Systematic Reviews.

[B35-behavsci-15-01005] Yen S., Ranney M. L., Tezanos K. M., Chuong A., Kahler C. W., Solomon J. B., Spirito A. (2019). Skills to enhance positivity in suicidal adolescents: Results from an open development Trial. Behavior Modification.

[B36-behavsci-15-01005] Yiu H. W., Rowe S., Wood L. (2021). A systematic review and meta-analysis of psychosocial interventions aiming to reduce risks of suicide and self-harm in psychiatric inpatients. Psychiatry Research.

