# Supplementary material for: Psychoeducation for Suicidal Behaviors in Inpatient Settings: A Scoping Review
Source: Behav Sci (Basel). 2025 Jul 23;15(8):1005. doi: 10.3390/bs15081005 (PMC12383175; doi:10.3390/bs15081005)
Supplement: Supplementary file 1 [file behavsci-15-01005-s001.zip › behavsci-3631117-supplementary.pdf]

Supplementary Material

Search string:

(psychoeducati* OR “psycho-edu*” OR “patient education” OR “patient teaching” OR "psychosocial education” OR "psycho-social education” OR "psychosocial intervention*” OR "psycho-social intervention*” OR "psychosocial therap*” "psycho-social therap*” OR "educational intervention*”) AND (inpatient* OR hospitaliz* OR hospitalis* OR “acute setting” OR “acute care setting” OR SPDC OR “psychiatry* ward*” OR “involuntary admission*” OR “compulsory admission*”) AND (“mental disorder*” OR “mental disease*” OR “psychiatric disorder*” OR “psychiatric disease*” OR psychosis OR psychotic OR schizophr* OR depressi* OR suicid* OR “personality disorder*” OR “self-harm” OR “self-injury” OR “bipolar disorder*” OR mania OR “obsessive-compulsive disorder*” OR “obsessive compulsive disorder*” OR “anxiety disorder*” OR “eating disorder*” OR “post-traumatic stress disorder*” OR “post traumatic stress disorder*” OR “substance use” OR “substance abuse”) AND (intervention* OR therap* OR treatment*) AND (trial* OR experimental* OR longitudinal)
